# Skip Regulates TGF-**β**1-Induced Extracellular Matrix Degrading Proteases Expression in Human PC-3 Prostate Cancer Cells

**DOI:** 10.1155/2013/398253

**Published:** 2013-05-20

**Authors:** Victor Villar, Jelena Kocic, Juan F. Santibanez

**Affiliations:** ^1^Laboratorio de Biología Celular, Instituto de Nutrición y Tecnología de los Alimentos, Universidad de Chile, 7810000 Santiago, Chile; ^2^Department of Biology, University of the Balearic Islands, Ctra Valldemossa, Km 7.5 , 07122 Palma de Mallorca, Spain; ^3^Laboratory for Experimental Haematology and Stem Cells, Institute for Medical Research, University of Belgrade, Dr. Subotica 4, P.O. Box 102, 11129 Belgrade, Serbia

## Abstract

*Purpose*. To determine whether Ski-interacting protein (SKIP) regulates TGF-**β**1-stimulated expression of urokinase-type plasminogen activator (uPA), matrix metalloproteinase-9 (MMP-9), and uPA Inhibitor (PAI-1) in the androgen-independent human prostate cancer cell model. *Materials and Methods*. PC-3 prostate cancer cell line was used. The role of SKIP was evaluated using synthetic small interference RNA (siRNA) compounds. The expression of uPA, MMP-9, and PAI-1 was evaluated by zymography assays, RT-PCR, and promoter transactivation analysis. *Results*. In PC-3 cells TGF-**β**1 treatment stimulated uPA, PAI-1, and MMP-9 expressions. The knockdown of SKIP in PC-3 cells enhanced the basal level of uPA, and TGF-**β**1 treatment inhibited uPA production. Both PAI-1 and MMP-9 production levels were increased in response to TGF-**β**1. The ectopic expression of SKIP inhibited both TGF-**β**1-induced uPA and MMP-9 promoter transactivation, while PAI-1 promoter response to the factor was unaffected. *Conclusions*. SKIP regulates the expression of uPA, PAI-1, and MMP-9 stimulated by TGF-**β**1 in PC-3 cells. Thus, SKIP is implicated in the regulation of extracellular matrix degradation and can therefore be suggested as a novel therapeutic target in prostate cancer treatment.

## 1. Introduction

Transforming growth factor *β*1 (TGF-*β*1) is implicated in the regulation of cell proliferation, differentiation, and migration, as well as extracellular matrix (ECM) production, apoptosis and tumorigenesis [1]. TGF-*β*1 is frequently overexpressed in carcinoma cells, including prostate cancer cells, and leads to paracrine stimulation and modification of cellular and extracellular matrix components of tumour microenvironment [2]. The urokinase-type plasminogen activator (uPA) system is thought to play a key role in cancer invasion and metastasis. uPA is a secreted serine proteinase that converts plasminogen to plasmin, a trypsin-like serine proteinase, which in turn can degrade a wide variety of ECM components and enable the tumour cells to penetrate the basement membrane, by facilitating cell migration and invasiveness [3]. uPA is tightly controlled by the specific serpin inhibitor PAI-1, which is also upregulated in cancer. PAI-1 can promote cell migration and angiogenesis independent of its effects on uPA-activated plasmin [4]. 

Matrix metalloproteinases (MMPs) have also been regarded as critical molecules in assisting tumour cells during metastasis. MMP-9, a member of the type IV collagenases, is known to influence cell proliferation, differentiation, angiogenesis, apoptosis and metastasis. After activation, MMP-9 is involved in proteolytic degradation of the ECM [5]. 

Increased expression of uPA, PAI-1, and MMP-9 reported in cancer has been related to poor tumour differentiation, invasive stage of cancer, poor patient prognosis, metastasis to secondary organs, and shorter survival time [3, 5–8]. In addition, in prostate cancer cells TGF-*β*1 stimulates the expression and activity of uPA, PAI-1, and MMP-9, resulting in a net increment of pericellular plasminogen activation, increased activation of MMP-9, and finally increased tumour cell invasion and metastasis [9, 10].

The signalling pathways by which TGF-*β* exerts its effects on cancer cell migration and invasion are gradually being elucidated. Recently, it has been reported that Ski-interacting protein (SKIP) interacts with Smad2,3 to enhance TGF-*β*-dependent transcription, suggesting its regulatory role in cell growth and differentiation through the TGF-*β* pathway [11]. SKIP is a well-conserved transcriptional adaptor protein that, depending on the cellular context, functions to recruit either activation or repression complexes to mediate multiple signalling pathways involved in the control of cell proliferation and differentiation [12]. However, its precise role in the stimulation of tumorigenesis by TGF-*β*1 is poorly understood. In this study, we investigated whether SKIP regulates the TGF-*β*1-induced extracellular matrix degrading system uPA/PAI-1 and MMP-9 expression in prostate carcinoma PC-3 cell line.

## 2. Material and Methods

### 2.1. Cell Culture

The human prostatic carcinoma cell line (PC-3) was obtained from the ATCC (Mannassas, VA) and cultured in DMEM : F12 (1 : 1) supplemented with 10% Foetal Bovine Serum. For TGF-*β*1 treatments, human recombinant TGF-*β*1 (R&D, Minneapolis, MN) was used at a 5 ng/mL. 

### 2.2. Antibodies

SKIP (C-15) and Smad2/3 ((FL-425): sc-8332) rabbit polyclonal antibodies were purchased from Santa Cruz Biotechnology (CA, USA). The p-Smad3 rabbit polyclonal antibody was purchased from Calbiochem, (Darmstadt, Germany). Anti-HA and secondary antibodies coupled to horseradish peroxidase were purchased from Sigma (Saint Louis MO, USA).

### 2.3. Plasmids and siRNA

HA-SKIP expressing plasmid was kindly provided by Dr. M. Hayman (Stony Brook University, USA). Human SKIP siRNA (sc-37164) and control siRNA (sc-37007) were purchased from Santa Cruz Biotechnology. uPA promoter (+2062 to +27) in luciferase reporter gene plasmid pGL2 basic (p-uPA-Luc) was a generous gift from Dr. Soishi Kojima from the Institute of Physical and Chemical Research (RIKEN), Tsukuba, Ibaraki, Japan. MMP-9 promoter construction was kindly provided by Dr. Takashi Kobayashi (Chiba University School of Medicine, USA). The promoter of PAI-1, p-800-luc (+71 to −800) was kindly provided by Dr. C. Bernabeu (CIB, Spain).

### 2.4. Transient Transfections and Reporter Gene Measurements

PC-3 cells seeded in 24 well plates (~2 × 10^4^ cells/well) were transfected with 500 ng/well of promoter luciferase constructions and 25 ng/well of SV40-*β*-Gal as internal control for transfection efficiency. Transfected cells were treated with TGF-*β*1 for 24 h. Firefly luciferase activity (Promega, Adison WI, USA) was standardized for *β*-galactosidase activity (Tropix, Bedford, MA, USA). For SKIP knockdown experiments, PC-3 cells grown in 6 well plates (~3 × 10^5^ cells/well) were transfected with 20 nM of SKIP siRNA or noneffective control siRNA.

### 2.5. Western Blot, Zymography, and RT-PCR Assays

Western blots were performed as described elsewhere [13]. MMP-9 and uPA activities were assayed in serum-free media conditioned for 24 h in cell cultures treated or not with TGF-*β*1. Conditioned media were subjected to SDS-PAGE zymography in gels containing 1 mg/mL gelatine for MMP-9 or casein-zymography assay for uPA, as reported previously [14].

Total RNA was obtained using Trizol and complementary DNA was generated by the SuperScript First-Strand Synthesis System for RT-PCR (Invitrogen, Carlsbad, CA, USA) using oligo (dT) primer. The following primers were used in this study: SKIP: 5′-GCG-CTC-ACC-AGC-TTT-TTA-CCT-GCA-CC-C-3′ Forward, 5′-CAC-GAC-AGG-CGC-AGG-AGG-AGA-AGC-3′ Reverse, 700 bp; MMP-9: 5′-GAG-ACC-GGT GAG-CTG-GAT-AG 3′ Forward, 5′ TCG-AAG-ATG-AAG-GGG-AAG-TG 3′ Reverse, 500 kb; PAI-1: 5′ CCA-CTT-CTT-CAG-GCT-GTT-CC 3′ Forward, 5′ GCA-GTT-CCA-GGA-TGT-CGT-AG 3′ Reverse, 350 kb; and GAPDH: 5′ACC-ACA-GTC-CAT-GCC-ATC-AC 3′ Forward, 5′ TCC-ACC-ACC-CTG-TTG-CTG-TA 3′ Reverse, 450 bp. Products were obtained after 30–35 cycles of amplification and electrophoresed in 1.2% agarose gels.

### 2.6. Statistics

Data are given as means ± SEM from at least three independent experiments. Asterisks (∗) denote significant differences at a value of *P* < 0.05. Horizontal brackets cover the groups that are being compared for statistical significance.

## 3. Results

### 3.1. SKIP Expression and TGF-*β*1-Induced uPA, MMP-9, and PAI-1 Production in PC-3 Cells

**Figure 1:**
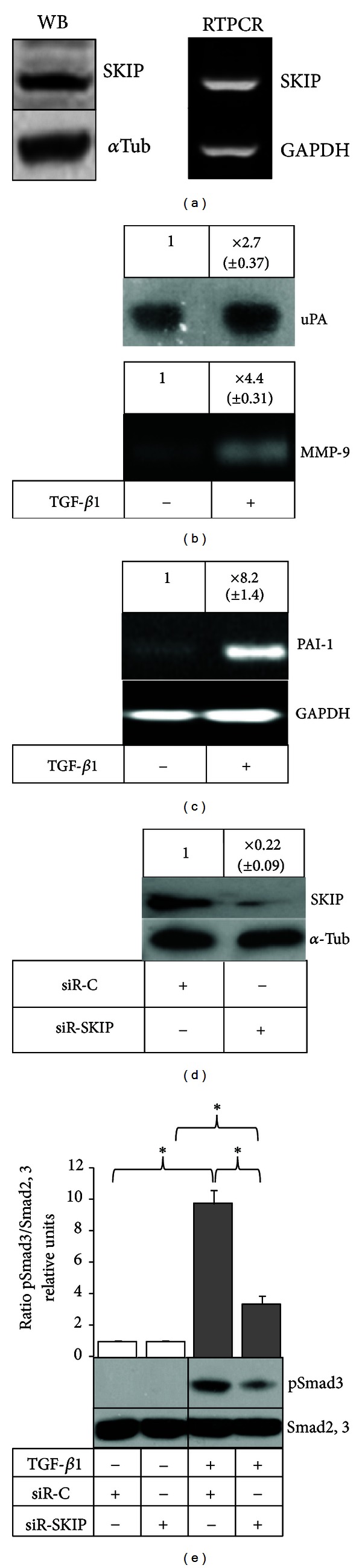
SKIP and TGF-*β*1-induced uPA, PAI-1, and MMP-9 expressions in PC-3 cells. The effect of SKIP knockdown on TGF-*β*1-induced Smad3 phosphorylation in PC-3 cells. (a) Expression of SKIP in PC-3 cells treated with TGF-*β*1 determined by Western blot and RT-PCR; *α*-tubulin and GADPH were used as a control of protein and cDNA loading, respectively. (b) Zymography analysis of uPA and MMP-9 in PC-3 cells treated with TGF-*β*1 for 24 h. (c) RT-PCR analysis for PAI-1 expression in PC-3 cells treated with TGF-*β*1 for 24 h. GADPH was used as housekeeper gene to verify the equal amount of cDNA in each sample. (d) SKIP siRNA knockdown in PC-3 cells determined by Western Blot. Cells were transfected with control siRNA (siR-C) or siRNA against SKIP (siR-SKIP). (e) Smad3 phosphorylation after 60 min of TGF-*β*1 treatment, determined by Western Blot in siR-C or siR-SKIP transfected cells.

First we examined the expression of SKIP in prostate cancer cells by western blot and RT-PCR analysis.  Figure 1(a) shows that PC-3 cells express both SKIP mRNA and protein. Next, we determined the capacity of TGF-*β*1 to modulate the production of extracellular matrix degrading enzymes, uPA and MMP-9, as well as uPA inhibitor PAI-1 in PC-3 cells. TGF-*β*1 greatly enhanced the production of uPA and MMP-9, as determined by zymography assays (Figure 1(b)), as well as the expression of PAI-1 mRNA transcript determined by RT-PCR (Figure 1(c)).

### 3.2. Knockdown of SKIP by siRNA in PC-3 Cells

To analyze whether SKIP participates in the effects of TGF-*β*1 on PC-3 cells, we subjected the cells to siRNA-mediated down-regulation of SKIP. As observed in Figure 1(d), the transfection with siRNA (20 nM) produced a dramatic depletion of SKIP expression compared with control siRNA transfected cells. We further analyzed the functionality of SKIP knockdown through the Smad3 activation by TGF-*β*1. As Figure 1(e) shows, the silencing of SKIP in PC-3 cells led to strong repression of TGF-*β*1-induced phosphorylation of Smad3 compared with the control.

### 3.3. SKIP Modulates uPA and PAI-1 Expressions

**Figure 2:**
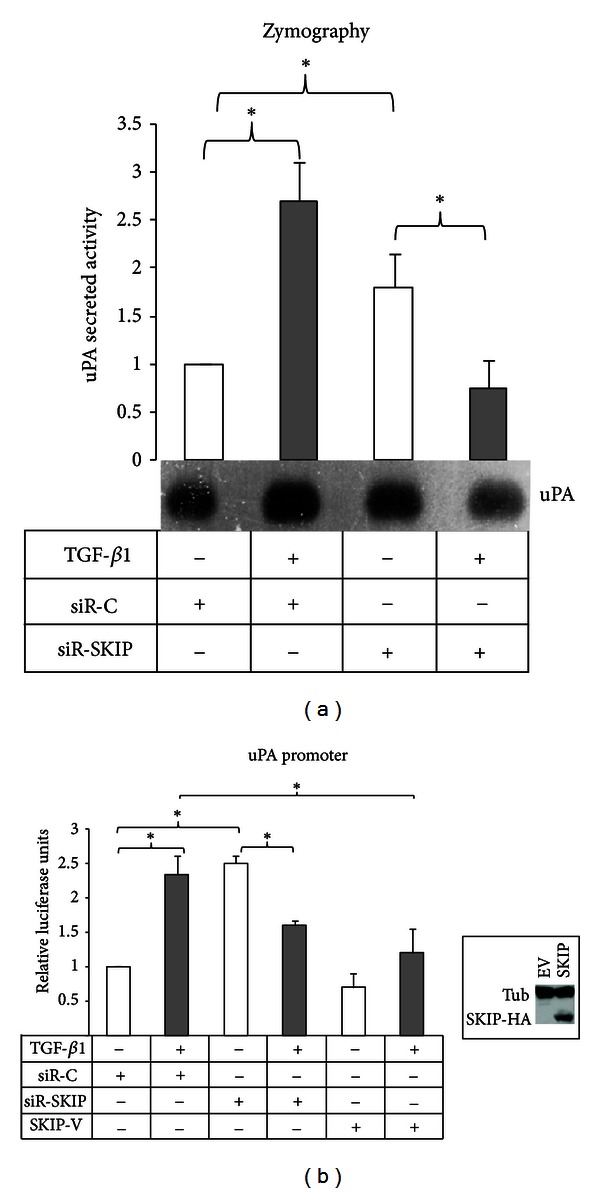
Effect of SKIP knockdown on TGF-*β*1-induced uPA expression. (a) Secreted uPA activity determined by zymography in the serum-free conditioned media of PC-3 cells transfected either with control siRNA (siR-C) or siRNA-SKIP (siR-SKIP), and treated or not with TGF-*β*1 for 24 h. (b) Transactivation of uPA promoter in PC-3 cells transiently transfected with uPA promoter construction and siR-C, siR-SKIP or SKIP-Ha tagged expressing vector (SKIP-V) and treated with TGF-*β*1 for 48 h. The correct expression of ectopic SKIP-HA in PC-3 cells was tested by Western Blot (insert).

To analyze whether SKIP is involved in TGF-*β*1-induced uPA expression in PC-3 cells, the activity of uPA secreted into the conditioned media of SKIP siRNA transfected cells was studied by zymography assay. In the SKIP-silenced cells, an enhanced production of the basal level of uPA was detected, while TGF-*β*1 treatment resulted in a dramatic inhibition of uPA expression, whereas in cells transfected with control siRNA TGF-*β*1 enhanced uPA production (Figure 2(a)). Intriguingly, the ectopic expression of SKIP also strongly inhibited TGF-*β*1-induced uPA promoter transactivation (Figure 2(b)).

**Figure 3:**
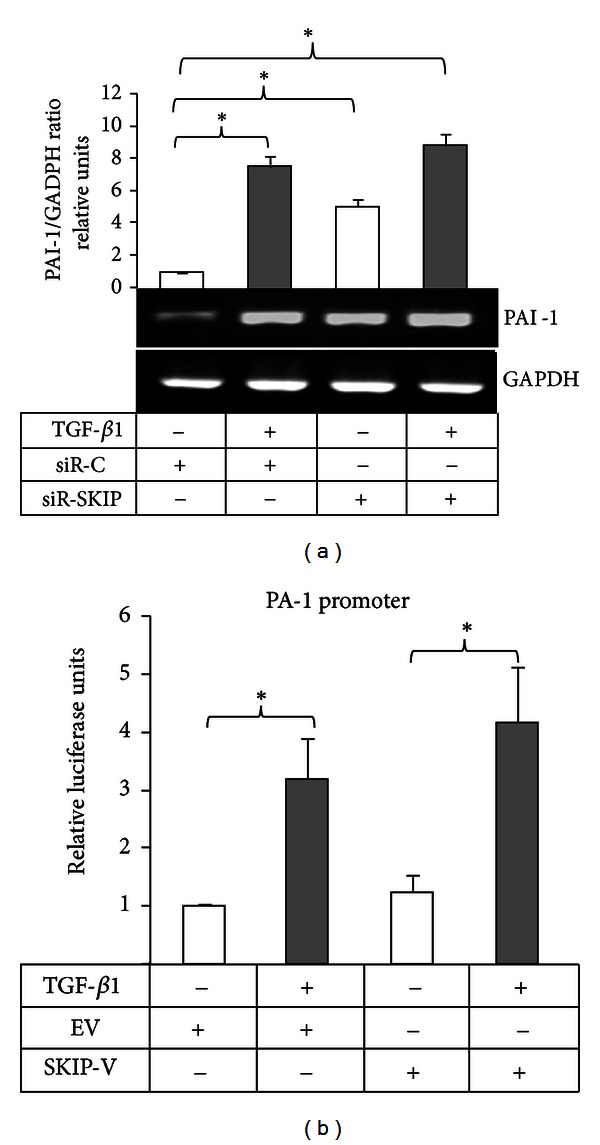
Effect of SKIP knockdown on TGF-*β*1-induced PAI-1 expression. (a) Expression of PAI-1 mRNA transcripts evaluated by RT-PCR in PC-3 cells transfected with either control siRNA (siR-C) or siRNA-SKIP (siR-SKIP) before and after stimulation (24 h) with TGF-*β*1. GAPDH was amplified as a control for the amount of cDNA present in each sample. (b) Transactivation of PAI-1 promoter in PC-3 cells is transiently transfected with empty vector (EV) as a control or SKIP-Ha tagged expressing vector (SKIP-V). MMP-9 promoter activity was assayed in cells unstimulated and stimulated with TGF-*β*1 for 48 h. *β*-Galactosidase was used as an internal control of transfection.

Additionally, SKIP knockdown enhanced the basal level of PAI-1 mRNA expression, which was slightly modified by TGF-*β*1 reaching the level of PAI-1 expression in control cells after TGF-*β*1 treatment (Figure 3(a)). Interestingly, when we determined the effect of the ectopic expression of SKIP on PAI-1 promoter, we did not find significant changes in the induction of the transactivation by TGF-*β*1 compared with control transfected cells (Figure 3(b)).

### 3.4. SKIP Silencing Enhances TGF-*β*1-Induced MMP-9 Expression

**Figure 4:**
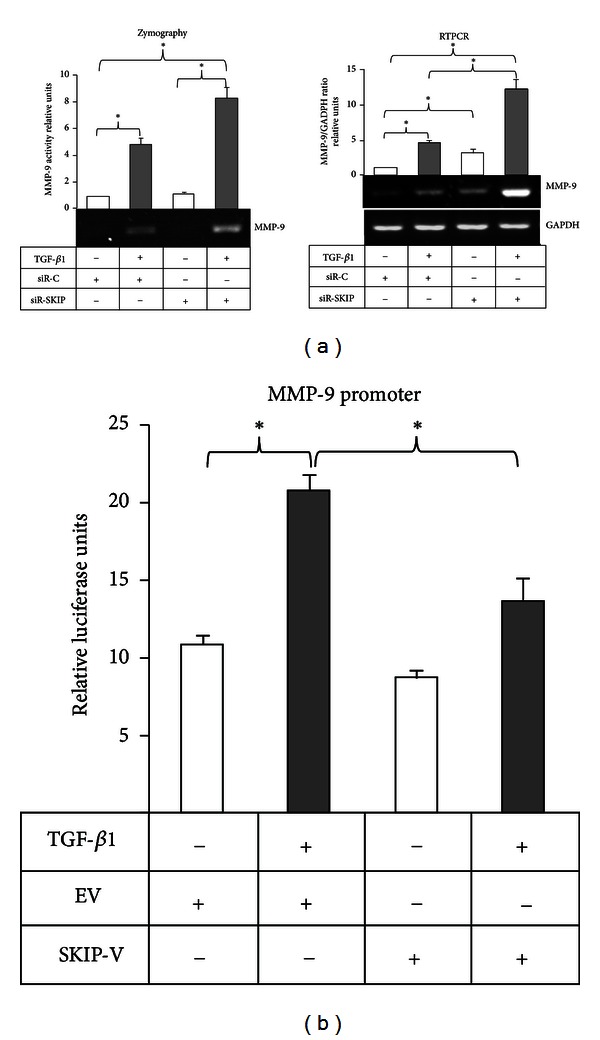
Effect of SKIP knockdown on TGF-*β*1-induced MMP-9 production. (a) MMP-9 zymography assay and RT-PCR analysis in PC-3 cells transfected with either siR-C or siR-SKIP and treated with TGF-*β*1 for 24 h. GAPDH was amplified as RT-PCR control for the amount of cDNA present in each sample. (b) Transactivation of MMP-9 promoter in PC-3 cells is transiently transfected with empty vector (EV) or SKIP-Ha tagged expressing vector (SKIP-V). MMP-9 promoter activity was assayed in cells unstimulated and stimulated with TGF-*β*1 for 48 h. *β*-Galactosidase was used as an internal control of transfection.

Our next goal was to analyze whether SKIP modulates TGF-*β*1-induced MMP-9 production. As shown in Figure 4(a), the stimulation of MMP-9 production by TGF-*β*1, determined by zymography, was strongly enhanced in SKIP-depleted cells relative to stimulated control cells. This result paralleled with that obtained by RT-PCR analysis, where the expression of the MMP-9 mRNA transcript was enhanced in SKIP siRNA-transfected cells under TGF-*β*1 treatment compared with control siRNA transfected cells. Furthermore, the effect of the ectopic expression of SKIP inhibited the stimulus of TGF-*β*1 on MMP-9 promoter activity when compared with control (Figure 4(b)).

## 4. Discussion

TGF-*β* is a multifunctional cytokine with an established role as a prometastatic agent in advanced cancer, and its expression has been negatively correlated with patient prognosis in malignant human prostate tumours [2]. The ability of TGF-*β* to stimulate invasion may represent an important contribution to the carcinogenic process in the prostate. Since TGF-*β*1 stimulates the invasiveness of tumour cells [1], it is important to discover which mechanisms control the intracellular signalling of this factor in transformed cells. Recently, the Ski-interacting protein (SKIP) has been shown to modulate Smads' activities in TGF-*β*1 signalling pathway [11], even though its role on TGF-*β*1-induced human cell malignance is not well elucidated yet. 

In the present work, we analyzed the role of SKIP in TGF-*β*1-stimulated expression of extracellular degrading proteinases uPA, MMP-9 and the inhibitor of uPA, PAI-1. These proteins are highly involved in tumour cell invasion and metastasis and are also known as poor prognosis markers in human cancer [3, 5–8]. As a cellular model we used the human prostate cancer PC-3 cell line, which was established from a prostatic adenocarcinoma metastasis in the bone, retaining *in vitro* features common to neoplastic cells of epithelial origin [14]. We observed that PC-3 cells express detectable levels of SKIP mRNA transcript and SKIP protein, and under TGF-*β*1 treatment the cells are induced to increase the production of uPA, MMP-9, and PAI-1 (Figure 1), which is in agreement with several previous reports [15–17]. 

The knockdown of SKIP increased basal production of uPA, while it decreased uPA production after TGF-*β*1 treatment (Figure 2). Given that SKIP depletion decreased Smad3 activation [13], and that we have previously reported Smad3 as essential for TGF-*β*1-induced uPA in transformed cells [18], we can speculate that the reduction of Smad3 activation by TGF-*β*1 may inverse the cell response to the growth factor, while in basal conditions the effect of SKIP on uPA expression may be independent of Smad3 signalling.

The activity of uPA is also regulated by the expression of its inhibitor PAI-1 [3, 4]. Interestingly, the downregulation of SKIP enhanced basal production of PAI-1 and this level was unaffected by TGF-*β*1 (Figure 3). A conceivable speculation for this result might be the involvement of SKIP in Retinoblastoma (Rb) inhibition. In hypophosphorylated state Rb is in complex with E2F, a cell cycle regulator, whereas when Rb is phosphorylated by CDKs, E2F is released and in free form acts as an inhibitor of PAI-1 expression [19]. Low expression of SKIP may keep Rb/E2F in complex, which could then result in the increment of PAI-1 expression independent of TGF-*β*1 stimulation. In addition, E2F is also a negative regulator of uPA expression, which could, in part, explain the enhanced basal level of uPA expression under SKIP depletion. Thus, SKIP depletion may affect basal uPA and PAI-1 expression independently of Smad3 in PC-3 cells.

Additionally, we observed that PC-3 cells increase the expressions of MMP-9 under TGF-*β*1 treatment and that the reduction of SKIP enhanced the TGF-*β*1-induced MMP-9 expression, while the ectopic expression of SKIP inhibited MMP-9 promoter transactivation (Figure 4). The effect of SKIP downregulation on TGF-*β*1-induced MMP-9 in part may be explained by the reduction of Smad3 activation, which could be necessary to regulate the adequate level of MMP-9 expression, whereas low levels of Smad3 activation may deregulate the control of MMP-9 expression in PC-3 cells in response to TGF-*β*1. This observation is supported by reports in which the missense mutations of the Smad3 gene or depletion of Smad3 in knockout mice showed increased MMP-9 production [20, 21]. 

## 5. Conclusions

The results presented here show that SKIP regulates the expression of uPA, PAI-1, and MMP-9 in response to TGF-*β*1 in PC-3 cells, implicating SKIP in the regulation of extracellular matrix degradation. Further studies should be performed in order to understand the magnitude of the possible role of SKIP in prostate cancer progression. Investigations committed to determining the level of SKIP expression in prostate cancer cells with different levels of malignance as well as analysis of clinical prostate cancer samples for its expression and distribution would be of high interest.
